# Differential Vascular and Non‐Vascular Binding Patterns of ACU193 and R‐Lecanemab in APP;hAPOE4 Mouse Brain Sections

**DOI:** 10.1002/alz70861_108078

**Published:** 2025-12-23

**Authors:** Martine B Grenon, Erika N Cline, Jasna Jerecic, Elizabeth Johnson, Eric Siemers, Cynthia A Lemere

**Affiliations:** ^1^ Brigham and Women's Hospital, Boston, MA USA; ^2^ Harvard Medical School, Boston, MA USA; ^3^ Maastricht University, Maastricht Netherlands; ^4^ Acumen Pharmaceuticals, Newton, MA USA

## Abstract

**Background:**

Therapeutic anti‐amyloid‐beta (Aβ) monoclonal antibodies (mAbs), lecanemab (Leqembi) and donanemab (Kisunla), have received FDA approval for treatment of early‐stage Alzheimer's disease (AD). In phase 3 clinical trials, ∼12–40% of patients developed Amyloid Related Imaging Abnormalities (ARIA), that may be due to mAbs binding vascular amyloid, the fibrillar deposits seen in cerebral amyloid angiopathy (CAA). To explore potential differences in binding profiles, we compared the vascular and non‐vascular binding of Aβ protofibril‐targeted mAb recombinant (r‐) lecanemab and Aβ oligomer‐targeted mAb ACU193.

**Method:**

Immunoreactivity of ACU193 and r‐lecanemab was characterized in serial sections of 20‐month‐old APPNL‐F/NL‐F;hApoE4 mice. Tissue was fixed for either 24‐hrs (*n* =3) or 30‐mins (*n* =3) to examine potential epitope masking associated with longer fixation times due to crosslinking. Serial sections were double‐labeled with anti‐amyloid antibodies (AAA) and fibrillar Aβ dye (Amylo‐Glo). A dilution series of 4 concentrations from 0.2 to 2.0 µg/ml was used. Non‐vascular (including plaque) binding was quantified in the cortex, and vascular binding was quantified in the cortex and cerebellum. Vascular labeling was further analyzed to determine the proportion that was fibrillar, based on colocalization with Amylo‐Glo.

**Result:**

In both fixation timepoints, the mAbs labeled non‐vascular Aβ in the cortex, with r‐lecanemab showing a higher percentage area of binding than ACU193. Mean mAb labelling was substantially higher in the 30‐min fixed tissue, with an average ∼4.8‐fold increase compared to 24‐hr fixation. ACU193 showed a concentration‐dependent increase at both fixation times, while r‐lecanemab showed concentration dependence only in 24‐hr fixed tissue and plateaued at 0.5 ug/ml in 30‐min fixed tissue. Cortical vascular staining was similar between mAbs, but r‐lecanemab showed greater fibrillar binding. In the cerebellum, the two mAbs had similar fibrillar binding, however r‐lecanemab stained an overall higher percent area than ACU193.

**Conclusion:**

ACU193 and r‐lecanemab differ in their concentration‐dependent binding, labeling of non‐vascular and vascular Aβ, fibrillar selectivity and regional patterns. Fixation time influenced antibody binding, particularly non‐vascular staining. Double‐label analyses with markers of Aβ, vasculature, neurons, and dystrophic neurites are underway to provide further insight into the localization of antibody binding. Funding_1R01NS136122‐01_CAL.

